# Cortactin is a sensitive biomarker relative to the poor prognosis of human hepatocellular carcinoma

**DOI:** 10.1186/1477-7819-11-74

**Published:** 2013-03-21

**Authors:** Gang Zhao, Zi-ming Huang, Ya-Lin Kong, Dong-Qing Wen, Yu Li, Li Ren, Hong-Yi Zhang

**Affiliations:** 1 Department of Hepatobiliary Surgery, Chinese PLA Air Force General Hospital, No.30 Fucheng Road, Haidian District, Beijing 100142, China; 2 Chinese PLA General Hospital & PLA Medical School, Beijing, China; 3 The Post-Graduate Institute of An Hui Medical University, Beijing, China; 4 Beijing Institute of Transfusion Medicine, Beijing, China; 5 Department of Pathology, Chinese PLA Air Force General Hospital, Beijing, China

**Keywords:** Hepatocellular carcinoma, Cortactin, Neoplasm invasiveness, Prognosis

## Abstract

**Background:**

Cortactin is an important regulator involved in invasion and migration of hepatocellular carcinoma (HCC). The aim of this study was to elucidate the forecasting role of cortactin in resectable HCCs.

**Methods:**

We compared the invasiveness and motility among liver epithelial cell line and HCC cell lines by using Transwell assay and wound healing assay. We further investigated the CTTN mRNA expression by real-time PCR. Next, 91 HCC and 20 normal liver tissue samples were detected by IHC and real-time PCR. Finally, we analyzed the clinicopathologic features and survival time of the HCC cases.

**Results:**

We identified that HepG2, LM3, and SK-Hep-1 had more invasiveness and motility (*P* <0.05). Compared with liver epithelial cell line, CTTN expression was higher in LM3, HepG2, and MHCC97-L (*P* <0.01) and lower in SK-Hep-1 (*P* <0.05). IHC examination showed cortactin expression was closely relative to TNM stage (AJCC/UICC), cancer embolus, and metastasis (*P* <0.01). Cortactin overexpression indicated a longer survival time of 52 ± 8.62 months and low expression of a shorter survival time of 20 ± 4.95 months (*P* <0.01). Cortactin examination has more predictive power in patients with Child-Pugh grade A and BCLC stage 0-B.

**Conclusions:**

Overexpression of cortactin is closely associated with poor human HCCs prognosis that caused by cancer embolus and metastasis. Cortactin and CTTN should be used for differentiating varieties of survival for patients after HCC resection.

## Background

Hepatocellular carcinoma (HCC) is one of the most common malignant tumors in the world [1]. In China, HCC has been one of the leading causes of cancer death despite remarkable improvement in diagnostic and therapeutic techniques [2]. Although the prognosis remains poor, there is no specific method to predict long-term survival time of HCC patients [3]. Thus, differentially subsequent treatment for HCC patients cannot be performed after initial operation. The point of view has been taken that poor prognosis of HCC is mainly because of the high incidence of hematogenous intrahepatic metastasis after initial treatment [4]. In essence, invasiveness of hepatocarcinoma cells play a crucial role in HCC metastasize and recurrence. Furthermore, cytoskeleton remodeling is required for tumor cell invasion and migration [5]. The molecular closely involved in cytoskeleton remodeling may be valid candidates which can predict prognosis of patients with cancer. Oligonucleotide array technology, a high-throughput method of monitoring gene expression, showed that the molecular signatures were apparently different between highly metastatic HCC cell lines and non-metastatic HCC cell lines. Among these candidates, CTTN, a gene related to tumor cell invasion and migration, for which the encoding protein is named cortactin, may be a potential molecular marker that can predict prognosis of cancer patients [6]. In addition, it has been well documented that CTTN is amplified in several other human cancers leading to cortactin overexpression [7,8]. Although stable overexpression of cortactin does not cause morphological changes or affect proliferation, it makes cells more motile and invasive [9]. It has been proved that if cortactin can be overexpressed in a non-metastatic HCC cell line it will increase cell motility and resulted in increased metastatic formation [6]. Conversely, cortactin knockdown can decrease tumor cell motility and invasion [6,10]. Forecasting role of cortactin has been investigated in head and neck squamous cell carcinomas and even in HCC [6,11]. As we know, however, no study has carried out follow-up on a series of cases with HCC. In this study, to investigate whether cortactin and CTTN could become a sensitive and specific biomarker to predicate HCC prognosis, we first selected five cell lines, including one liver epithelial cell line and four HCC cell lines, to detect their ability of invasion and migration. We then compared their expression level of CTTN mRNA. We also collected patients who were performed hepatectomy and examined their tissue samples. Cortactin and CTTN mRNA were examined respectively. To confirm whether cortactin and CTTN expression are related to the survival of HCC patients, we followed up this series of cases and compared the survival data of different expression levels.

## Methods

### Cell culture and liver tissue

The liver epithelial cell line, QSG-7701, and HCC cell lines, HepG2, MHCC97-L, and SK-Hep-1, were obtained from the Beijing Institute of Transfusion Medicine. Another HCC cell line, LM3, was obtained from the Chinese PLA General Hospital. All the cells were maintained in DMEM (H) supplemented with 10% fetal bovine serum (FBS). Ninety-one random sampled HCC patients (22 women, 69 men; mean age, 54 years) were studied retrospectively. The patients had been recruited in the PLA Air Force General Hospital from 2002 to 2009. All the patients have been performed hepatectomy for resectable hepatocellular carcinoma. No radiotherapy and chemotherapy were undergone before the operation. The other 20 hepatic haemangioma cases were randomly sampled as the control group. The final pathological diagnoses of 111 cases were definite. Ninety-seven tissue blocks were acquired and refrigerated in liquid nitrogen right after removal out of the body, including 77 HCC tissues and 20 normal liver tissues adjacent to haemangioma. In 91 HCCs, 14 tissues were not acquired to be refrigerated for the reason of too small tumor size or inconvenient during operation. Patients and disease characteristics are shown in Table 1. Sections from 91 HCC histological paraffin-embedded specimens were used for cortactin immunohistochemistry. Seventy-seven HCC tissue blocks and 20 normal liver tissue blocks were analyzed by real-time PCR to investigate CTTN mRNA expression level. Written informed consent was obtained from all patients, and this study was approved by the Ethics Committee of Chinese PLA Air Force General Hospital.

**Table 1:** Cortactin immunohistochemical detection in 91 HCCs

	**Cortactin IHC score**		***P***
	**0-3**	**4-6**	
Cases (*n*)	43	48	
Gender			0.846
Male	33	36	
Female	10	12	
Mean age (years)^a^	52.81 ± 1.74	54.46 ± 1.68	0.667
HBsAg			0.265
(−)	9	15	
(+)	34	33	
AFP (μg/L)^a^	276.79 ± 57.33	319.6 ± 61.57	0.614
Liver cirrhosis			0.523
No	10	14	
Yes	33	34	
Maximal size (mm)^a^	6.64 ± 0.84	7.76 ± 0.66	0.531
Liver capsule integrity			0.054
Complete	34	29	
Incomplete	9	19	
Tumor nodules (*n*)			0.084
Single	34	30	
Multiple	9	18	
Cancer embolus or metastasis			0.000^b^
No	40	21	
Yes	3	27	
Edmondson classification			0.375
I-II	21	19	
III-IV	22	29	
TNM stage (AJCC/UICC)			0.000^b^
I-II	32	15	
III-IV	11	33	

^a^Mean ± SE.

^b^*P* <0.01.

### The determination of clinical stage

Tumor size and number, liver function test, prothrombin time, performance status, and cancer-related symptoms were investigated. If esophagogastric varices, ascites, splenomegaly and a platelet count <100,000 /mm^3^, the portal hypertension was defined. All patients underwent serum α-fetoprotein (AFP), abdominal ultrasound, and four-phase dynamic computed tomography (CT) examination. In cases with another intrahepatic nodule not typical of HCC by dynamic CT and AFP, evaluations were made by dynamic magnetic resonance imaging (MRI) or positron emission tomography (PET)-CT. The biopsy was applied when imageology examination could not make a final diagnosis. The chest CT was carried out when the patient had positive present of diagnostic chest X-ray radiology which is as a routine examination for all patients. A bone scan was necessary when the patient have chief complaint of osteodynia. The hepatic functional reserve was evaluated by Child-Pugh grade system which was classified as A, B, and C. BCLC stage system was applied to determine the clinical stages. HCC patients were classified into five stages including: 0, very early; A, early; B, intermediate; C, advanced; or D, terminal. Final HCC stages were determined after operation, histopathology, and a follow-up examination [12].

### Transwell invasion assay

To hydrate the matrigel layer, the upper chamber of a transwell cell culture insert (Corning 3422) was filled with 100 μL DMEM(H) medium without FBS and incubated at 37°C for 1 h. Cells were suspended with serum-free DMEM(H) medium to the concentration of 5 × 10^5^ cells/mL. Filled 200 μL cell suspension to upper chamber and 600 μL DMEM(H) medium with 10% FBS to the bottom chamber. The inserts were placed in the cell culture incubator at 37°C for 24 h. The cell was fixed by placing each insert into 90% ethanol and the cell was stained with 1% crystal violet for 30 min. Five high power fields (×100) were randomly selected in each chamber to observe the cells and Cell Counter software was used to count the stained invaded cells. Each experimental group was repeated three times.

### Wound healing assay

Cells were seeded into 24-well tissue culture plate in a density that reached 70% to 80% confluence as a monolayer after 24 h of growth. The monolayer was scratched with a pipette tip across the center to create a cross in each well. The well was washed twice with medium to remove the detached cells. The cells were grown for additional 96 h in fresh medium. Four views of each well were documented and the gap distance was quantitatively evaluated using WCIF ImageJ software. Each experimental group was repeated three times.

### Immunohistochemical staining

Labeled streptavidin-biotin peroxidase complex method was used [13]. Immunohistochemistry with cortactin (Cortactin Ab, 1:50; Cell Signaling) was performed on sections of formalin-fixed (7.5% buffered formalin) and paraffin-embedded representative tumor tissues from all 91 carcinomas. Tissue blocks were cut to 4 μm, then mounted on silicone-coated slides, deparaffinized with xylene, 100% ethanol and 95% ethanol by turns, and heated in 10 mM sodium citrate buffer (pH 6.0) using a microwave oven for 10 min at 95°C. Before primary antibody incubation, incubated sections in 3% hydrogen peroxide and blocked sections with 5% goat serum. Primary antibodies were incubated overnight at 4°C. Biotinylated secondary antibody and ABC avidin/biotin method was used for visualizing positive reactions. After ABC reagent was incubated, the sections were developed with 3, 3'-diaminobenzidine and counterstained with hematoxylin. The sections were washed, dehydrated, and mounted as routine.

### Immunohistochemical scoring

For each stained section, the fraction of the immunostained cells was recorded, and the staining intensity was then initially categorized according to arbitrarily predefined criteria into three ranks, including: 0, very weakly or weakly positive; 1, moderately positive; and 2, strongly positive. Five high power fields (×400) were randomly selected in each section, and no fewer than 1,000 tumor cells were observed. The percentage of positivity was calculated. The percentage was scored from 0 to 4 according to the percentage of positive cells (0, 0-5%; 1, 6-25%; 2, 26-50%; 3, 51-75%; 4, 76-100%). Positive percentage score plus stain intensity score were the final score of each section [14,15]. The examiners were blinded to patients’ clinical and histological (HE staining) profile. Two investigators evaluated the staining levels independently, after which any discordant evaluations were adjusted by connected microscopes and scored jointly.

### Single strand cDNA synthesis

Trizol (Invitrogen) was used to isolate total RNA from cultured cells and tissue blocks following the manufacturer’s instruction. RNA integrity was confirmed spectrophotometrically and by electrophoresis on 1% agarose gels [16]. For cDNA synthesis, 2 μg total RNA of a sample were reverse-transcribed in a final reaction volume of 25 μL containing M-MLV (Promega), random hexamers, dNTP, RNase inhibitor (TaKaRa). The reverse transcription reaction was performed under the following conditions: 70°C for 5 min followed cooling the tube immediately on ice; then at 25°C for 10 min, 37°C for 1 h, and 85°C for 15 min.

### Real-time PCR

CTTN and GAPDH (glyceraldehyde phosphate dehydrogenase) gene were amplified by RT-PCR (reverse transcription-polymerase chain reaction) from one HCC samples. The products of each gene were respectively inserted into pMD18-T vector to build standard curves. The amount of standard substance copies was confirmed spectrophotometrically. The primer set 5’-TGAGTGTGTGTTCTTCCCCAAG-3’ (forward) and 5’-CACGTGACCTTCTGGAAAGACA-3’ (reverse) [6] was used for CTTN. For standardization among the samples, GAPDH was used as internal reference gene and the expression of GAPDH in each sample was quantified by primer set 5’-AGAAGGCTGGGGCTCATTTG-3’ (forward) and 5’-AGGGGCCATCCACAGTCTTC-3’ (reverse). 2 × SYBR Premix Ex Taq (TaKaRa DRR041) was used and the reaction was performed at least three times for each sample. For the signal detection, Eppendorf Mastercycler ep realplex was programmed to an initial step of 10 s at 95°C and 40 thermal cycles of 5 s at 95°C, 25 s at 58°C, and 15 s at 72°C. After PCR reaction, melting curve reaction was performed. For analyzing the correlation between CTTN mRNA expression level and HCC invasiveness, the CTTN relative value to housekeeping gene GAPDH was calculated. One hemangioma-neighboring liver tissue sample became the 1× sample, and all the other quantities were expressed as an *n*-fold difference relative to this tissue.

### Statistical methods

Data are expressed as means ± SE. The level of cortactin mRNA in HCC cell lines and liver epithelial cell line was compared using unpaired *t*-test. The correlations between cortactin expression and clinicopathological features were analyzed by Pearson χ^2^ test and unpaired *t*-test. To compare the difference of CTTN mRNA relative magnitude, the variance analysis and Kruskal-Wallis rank sum test was used. Cancer-specific survival time was calculated according to the Kaplan-Meier method. The log-rank test was used to compare the groups. *P* <0.05 was considered significant. All these analyses were performed using SPSS 17.0.

## Results

### Invasiveness and motility

**Figure 1:**
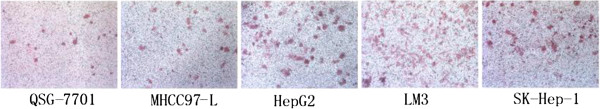
Decoration of puncture cells by five cell lines (100 × magnifications).

**Figure 2:**
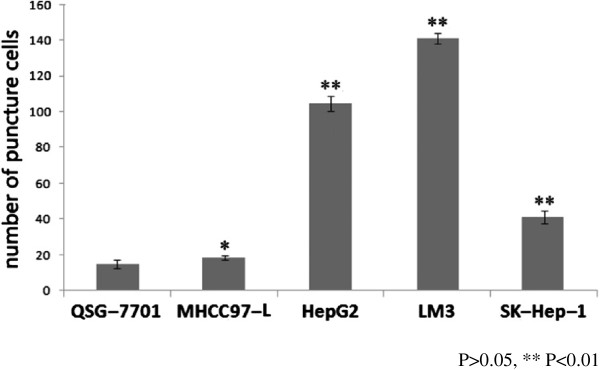
**Counted number of puncture cells with five cell lines.** QSG-7701 was the control cell lines, *P >0.05, **P <0.01.

**Figure 3:**
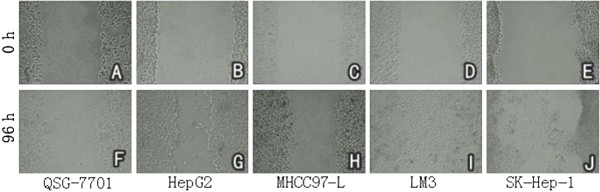
Gap distance of scratch wound healing assay with five cell lines (50× magnification).

**Figure 4:**
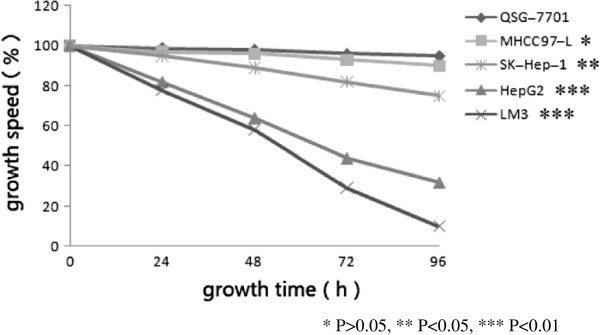
**Diversify of scarification area with growth of five cell lines.** QSG-7701 was the control cell lines, *P >0.05, **P <0.05, ***P <0.01.

Compared with QSG-7701, a non-cancerous liver epithelial cell line, the HCC cell lines HepG2, LM3, and SK-Hep-1 had more invasiveness (*P* <0.01). But not all HCC cell lines had significant invasiveness such as MHCC97-L (*P* >0.05). (Figures 1 and 2) The metastasis ability of HepG2, LM3, and SK-Hep-1 were also significantly superior to QSG-7701 (*P* <0.01, *P* <0.01, *P* <0.05). MHCC97-L had poor metastasis ability (*P* >0.05) (Figures 3 and 4).

### CTTN quantity method

The CTTN quantitative standard curve had finer linear relation when copy number of standard products was 1.6 × 10^3^-1.6 × 10^7^, as well as GAPDH of 1.6 × 10^4^-1.6 × 10^7^. R^2^ of both curves were >0.99. Single peak in the melt curve identified specific PCR amplification. CTTN specific peak localized at 88.0°C and GAPDH at 87.2°C. Preliminary test was performed to evaluate the repeatability of CTTN quantity method before the final detection. Five specimens of HCC were detected at 4 different days with three repetitions each run. Threshold cycle values (Ct) were collected to statistics the data. The outcome showed that the repeatability of each specimen had no significant difference within run and between days (data not shown).

### CTTN expression in HCC cell lines

**Figure 5:**
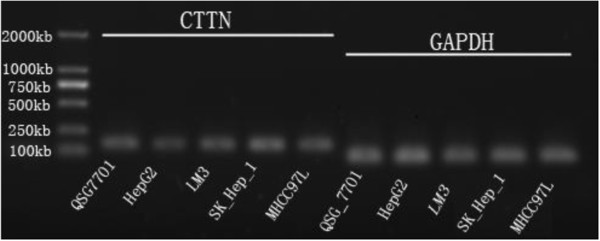
Electrophoresis of CTTN and GAPDH amplified products with five cell lines.

**Figure 6:**
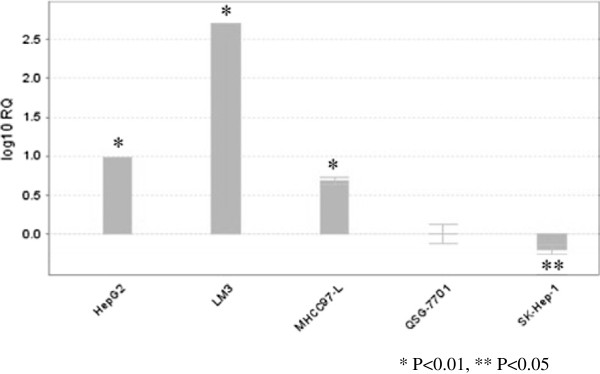
**CTTN relative expression with five cell lines (logarithm).** QSG-7701 was the control cell lines, *P <0.01, **P <0.05.

The amplification products of each cell lines were electrophoresed in 1.5% agarose gel to indicate correct amplification (Figure 5). ALL HCC cell lines have different expression levels of CTTN mRNA. Compared with QSG-7701, the CTTN mRNA of HepG2, LM3, and MHCC97-L overexpressed to 4.86-517.75 times (*P* <0.01). SK-Hep-1 had low expression of 0.63 times (*P* <0.05) (Figure 6).

### Cortactin expression in HCC tissues

**Figure 7:**
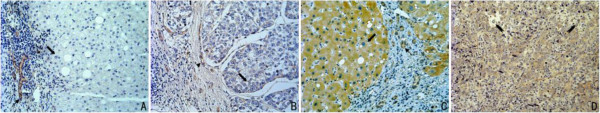
**Cortactin expression in HCCs (400× magnification).** (**A**) Bile duct epithelial cells (thin arrow) were strong positive, whereas HCC tissue (thick arrow) was negative. (**B**) Vascular endothelial cells (arrowhead) were positive with HCC tissue of weak positive expression. (**C**) HCC tissue showed strong positivity with diffuse staining of cytoplasm. (**D**) HCC tissue showed strong positive. Cancer cells were in a state of chaos with severe morphosis. Cancer embolus (double tail arrow) in portal vein can be seen.

Among the 91 HCC tissues, cortacin positivity was observed in most sections. Only two cases were completely negative. Forty-three cases scored 0 to 3 and 48 cases scored 4 to 6. In positive sections, cortactin immunoreactivity was detected on cytoplasm and showed diffuse staining. On positive cells, the cell membrane also showed positive staining with similar intensity of cytoplasm (Figure 7). Non-tumorous hepatocyte was almost negative regardless of chronic hepatitis or cirrhosis. However, bile duct epithelial cells and vascular endothelial cells always stained strongly. This could ensure the correct staining and the following scoring.

### Cortactin and clinicopathological features

No significant difference was detected in gender, age, HBsAg, AFP, liver cirrhosis, tumor size, Edmondson classification, number of tumor nodules, and liver capsule integrity (*P* >0.05). On the contrary, statistical difference was detected in the cases with cancer embolus in portal vein or distant neoplasm metastasis (*P* <0.001) (Table 1).

### CTTN expression in HCC tissues

**Figure 8:**
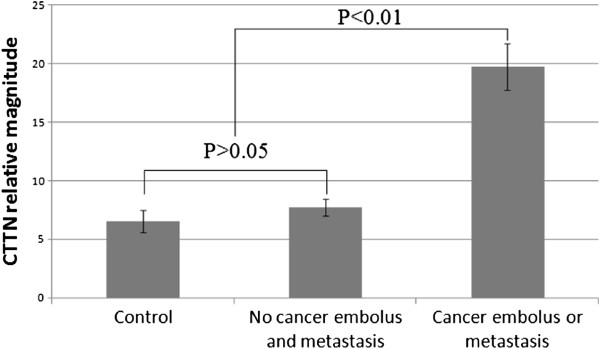
CTTN relative magnitudes of no cancer embolus and metastasis group, cancer embolus or metastasis group, and control group.

According to IHC analysis, we grouped the 91 HCC cases by whether cancer embolus and metastasis could be observed. Statistical results showed, although the CTTN relative values of no cancer embolus and metastasis group (N), cancer embolus or metastasis group (Y), and control group (C) were tested normally distributed, the heterogeneity of variance existed between groups (*P* <0.001). Kruskal-Wallis H test showed that the relative magnitude was significantly different between three groups. Paired comparison indicated the relative magnitude of Y group was much higher than that of the other two groups (*P* <0.001). There was no statistical significance between the N and C groups (*P* >0.05) (Table 2, Figure 8).

**Table 2:** CTTN relative magnitude of the three groups

**Group**	***n***	$\bar{x}$**± SE**	**Minimal value**	**Maximal value**	**95%CI**
Control	20	6.54 ± 0.96	1.00	15.72	4.53-8.56
No cancer embolus and metastasis	52	7.75 ± 0.70	1.15	20.35	6.34-9.16
Cancer embolus or metastasis	25	19.73 ± 2.02	2.99	42.47	15.56-23.90

Levene test of homogeneity of variance: F = 10.57, *P* <0.001; Kruskal-Wallis H test: H = 31.13, *P* <0.001.

### Survival analysis

**Figure 9:**
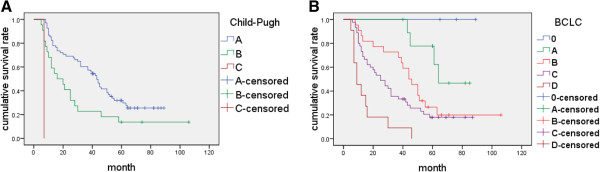
**Survival analyses by Child-Pugh grade and BCLC stage.** (**A**) Kaplan-Meier curves of 91 HCC patients grouped by Child-Pugh grades A, B, and C (*P* <0.001); (**B**) Kaplan-Meier curves of 91 HCC patients grouped by BCLC stages 0, A, B, C, and D (*P* <0.001).

The general median survival time of this group of 91 HCC patients was 37 ± 6.62 months. The authors carried out survival analysis by the classification of Child-Pugh grade. The result showed that grade A and B have a different median survival time of 43 ± 2.89 and 16 ± 5.28 months (*P* <0.001). In this group of data, only one patient was identified with grade C and survived 7 months after the initial diagnosis and operation (Figure 9A). According to BCLC stage system, three (3.3%) patients were identified into group 0, 10 (11.0%) into group A, 22 (24.2%) into group B, 45 (49.5%) into group C, and 11 (12.1%) into group D. No patients died of HCC in group 0. The median survival times of groups A, B, C, and D were 64, 44 ± 4.69, 25 ± 4.70, and 9 ± 2.75 months, respectively. The significant deviation was found between the four groups (*P* <0.001). The median survival time of group 0 and the standard error of group A could not be calculated for the censored data (Figure 9B).

**Figure 10:**
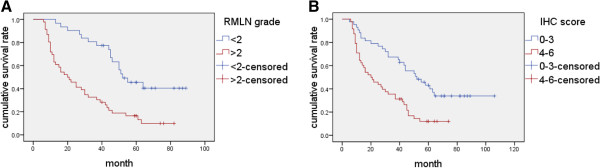
**Survival analyses with two kinds of grouping method.** (**A**) Kaplan-Meier curves of 77 HCC patients with CTTN RMLN grades of <2 and >2 (*P* <0.001); (**B**) Kaplan-Meier curves of 91 HCC patients with cortactin IHC score ranks of 0–3 and 4–6 (*P* <0.001).

Survival analysis was performed by two grouping methods, including by according to the CTTN relative magnitude and cortactin IHC score. For the convenience of statistical analysis, the natural logarithm of relative magnitude (RMLN) of each HCC specimen was calculated. The critical value was set at 2. The difference between these two groups was detected (*P* <0.001) with median survival time of 52 ± 8.17 months (RMLN <2) and 20 ± 4.24 months (RMLN >2) (Figure 10A). With IHC score grouping, it had also shown difference between the 0–3 and 4–6 groups (*P* < 0.001). The median survival time was 52 ± 8.62 months and 20 ± 4.95 months, respectively (Figure 10B).

## Discussion

Recent research has shown that several tumor markers in serum are applied for the detection and forecasting on HCCs. The recommended screening strategy for patients with cirrhosis includes the determination of serum α-fetoprotein (AFP) levels to detect HCC at an earlier stage. However, AFP is not satisfactory for the poor sensitivity and specificity. In addition to AFP, Lens culinaris agglutinin-reactive AFP (AFP-L3), des-γ-carboxy prothrombin (DCP), glypican-3 (GPC-3), osteopontin (OPN), and several other biomarkers (such as squamous cell carcinoma antigen-immunoglobulin M complexes [SCCA-IgM], alpha-1-fucosidase [AFU], chromogranin A [CgA], human hepatocyte growth factor, insulin-like growth factor) have been proposed as markers for the detection of HCC. Although these markers have their diagnostic and prognosis roles, none of them is optimal [17]. When used together, the DCP and AFP assays increase the sensitivity of detecting HCC in >85% of patients. The specificity of the DCP assay appears to be superior to that of AFP [18]. Recently, there has been attention given to DCP because of its role in detecting HCC metastases and recurrence. The serum DCP level correlates with the presence of vascular invasion or intrahepatic metastases. Furthermore, DCP has been reported to be an independent prognostic factor for recurrence and survival after hepatic resection and other treatment. High DCP levels reflect the biologic aggressiveness and progression of HCC [18].

In addition to serum markers, the tremendous advances in the research on HCC tissues have shown expression difference in several genes. The most relevant result is the identification of an HCC-related index consisting of 13 genes (TERT, IGF2, GJB2, TEF, TIAM1, CXCL12, TOP2A, A2M, PLG, ARF, PDGFRA, MKI67, and THBS1), which allowed the diagnosis of HCC with a high sensitivity and a specificity of 100%. Therefore, molecular analysis was shown to be a valuable tool for diagnosing HCC [16]. Another research has reported that 12 genes were expressed differently in initial HCC versus dysplastic nodules: five were overexpressed (TERT, glypican-3, gankyrin, survivin, TOP2A) and seven were subexpressed (LYVE1, E-cadherin, IGFBP3, PDGFRA, TGFA, cyclin D1, HGF) [19]. The same applies for biomarkers such as VEGF, angiopoietin 2, or the proto-oncogene c-Kit. They can refine prognostic prediction within statistical modeling but cannot yet be incorporated into assessment of an individual patient [12].

Cortactin was originally identified as one of the major substrates for the protein kinase Src [20]. Cortactin gene, CTTN (formerly designated EMS1), was identified to be amplified in several human cancers leading to cortactin overexpression [21]. It localizes in the 11q13 that is frequently overexpressed in breast and head and neck cancers and is tied to poor prognosis [22]. The amplification of 11q13 includes several molecular markers frequently associated with higher pathological stage, lymph node and distant metastasis, and decreased survival [23-25]. Due to the ubiquitous presence in cell motility structures, such as lamellipodia and invadopodia [21,26], cortactin generates a great deal of interest in the role in tumor invasion. Cortactin contains a proline-rich region with c-Src tyrosine phosphorylation sites and a SH3 domain at the COOH terminus [27]. It also contains an N-terminal acidic region that binds to the Arp2/3 complex [28]. Phosphorylation binding sites and SH3 domain are necessary for both activation and regulation of Arp2/3-complex-mediated branched actin assembly [29]. In addition to directly regulating actin assembly, cortactin also activates the neural Wiskott-Aldrich syndrome protein (N-WASP), one of the strongest activators of the Arp2/3 complex [30,31]. By using transwell migration [9], wound closure [32], and single cell motility, it has been demonstrated that cortactin can enhance cell motility. On the other hand, siRNA against cortactin inhibits cell motility [33,34]. Resent research suggest that cortactin may regulate endocytosis of integrins and growth factor receptors [35] or secretion of proteases [36] and extracellular matrix (ECM). These regulations have effects on cell motility, which dependent cell context.

Evidence shows that invasion is the most important factor which can raise recurrence rate of HCC [37]. Diffusion within or outside liver though portal vein, which originated from invasion of malignant cells, indicates poor prognosis of HCC [38]. Current studies demonstrate that overexpression of cortactin was closely associated with intrahepatic metastasis in human HCC and was a sensitive marker for HCC with intrahepatic metastasis [6]. But now it is lack of clinical research concerning the relation between CTTN expression level and HCC survival. Furthermore, to the best of our knowledge, no molecule markers can predict HCC prognosis independently and precisely. For this reason, we applied IHC and real-time PCR to detect cortactin expression in both protein and mRNA level with the intention of developing an efficacious approach to predict prognosis of HCC patients.

First, we compared five cell lines upon invasiveness and motility. The results showed that the invasion ability of three HCC cell lines were superior to the liver epithelial cell line. The motility of LM3, HepG2, and SK-Hep-1 were superior to QSG-7701, whereas MHCC97-L was not higher than that of QSG-7701. Interestingly, compared with liver epithelial cells, the CTTN expression of HCC cells may be overexpression, such as LM3, HepG2, and MHCC97-L, or low expression, such as SK-Hep-1. These results indicate that although CTTN overexpression may signify the higher invasiveness and motility, low expression may not be the sign of lower invasiveness and motility. The phenomenon was also observed in the examination of human tissue samples.

Subsequently, we detected cortactin IHC reactivity of HCCs. As our data showed, cortactin immunohistochemistry score was significantly associated with cancer embolus in portal vein and distant neoplasm metastasis, by both separately analysis and jointly analysis. This result is accordant with that of the other authors [6,38,39]. Apart from cancer embolus and distant metastasis, it is worth noting that liver capsule integrity may be the latent correlation factor to cortactin expression, though the *P* values were 0.054. A recent report has shown that extracapsular penetration is an important prognostic factor in human hepatocellular carcinoma [40]. In fact, if hemorrhage occurs because of spontaneous rupture in HCC or tumor cells break through pseudocapsule and form satellite lesion, the capsule integrity would definitely be destructed. The AJCC/UICC TNM stage system emphasize the role of neoplasm invasion and metastasis. We suppose that is the reason of correlativity between TNM stage and cortactin immunohistochemical reaction. Thus higher cortactin immunohistochemical score may imply higher TNM stage of HCC, which is significant to the clinical decision-making for the treatment to HCC.

This series of data also showed that CTTN mRNA expression level of cancer embolus or metastasis group (Y) was higher than no cancer embolus and metastasis group (N) and control group (C). But we found no difference between the N and C groups. This finding may indicated that CTTN mRNA expression level is not upregulated in non-metastatic HCCs. Just as the detected results from HCC cell lines, CTTN expression in MHCC97-L, a low invasiveness cell line, had the mildest upregulation. SK-Hep-1 is a special cell line that cannot form solid tumor. Its CTTN mRNA expression was not upregulation. We supposed that it is because cortactin have the role in regulation of the adhesion between cells. It has been reported that the adhesion regulated by cortactin may be relative to CD44, an important molecule to signal other cell surface receptors involved in regulating cell motility and invasion [33]. Thus, low expression of cortactin cannot promote the efficient adhesion which cannot lead HCC cells to format solid tumor, just as the biological behavior that SK-Hep-1 has.

In clinical practice, accurately predicting prognosis as far as possible is very important to the therapeutic decision-making for HCC patients. To investigate the prediction power by cortactin and CTTN, we bring into cortactin immunohistochemical score and natural logarithm of CTTN mRNA relative magnitude (RMLN). The survival analysis showed that both methods could distinguish the variance between high and low cortactin expression. Moreover, the median survival time was similar to each other. This congruous result further demonstrated the forecasting role of cortactin in both mRNA and protein level.

**Figure 11:**
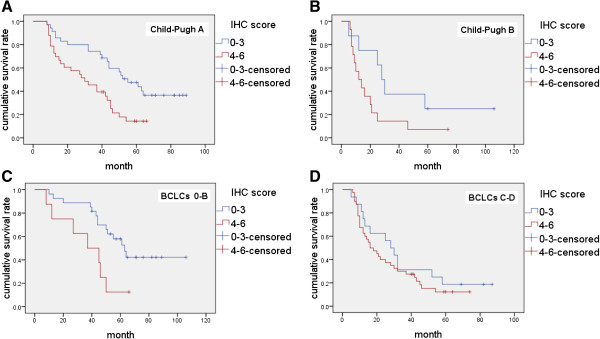
**Survival analyses stratified by Child-Pugh grade and BCLC stage.** (**A**) Kaplan-Meier curves of 68 HCC patients (stratum Child-Pugh A) with cortactin IHC score ranks of 0–3 and 4–6 (*P* <0.01); (**B**) Kaplan-Meier curves of 22 HCC patients (stratum Child-Pugh B) with cortactin IHC score ranks of 0–3 and 4–6 (*P* = 0.071); (**C**) Kaplan-Meier curves of 35 HCC patients (stratum BCLC 0-B) with cortactin IHC score ranks of 0–3 and 4–6 (*P* = 0.015); (**D**) Kaplan-Meier curves of 56 HCC patients (stratum BCLC C-D) with cortactin IHC score ranks of 0–3 and 4–6 (*P* >0.05).

To preclude the confounding factor that hepatic functional reserve contributing to the survivals, we performed a survival analysis stratified by Child-Pugh grade. The results showed that in the stratum with Child-Pugh grade A, the median survival time was 55 ± 7.38 months in the patients whose cortactin IHC score was 0–3; but it was only 30 ± 7.18 months in the patients whose cortactin IHC score was 4–6. The difference between the two cortactin expression levels was significant (*P* <0.01) (Figure 11A). In stratum with Child-Pugh grade B, the median survival time was 28 ± 3.54 months and 12 ± 3.74 months in levels with cortactin scores of 0–3 and 4–6, respectively. However, the difference between these two cortactin expression levels was not significant (*P* = 0.071) (Figure 11B). Hence the authors supposed that the decrease of hepatic functional reserve may reduce the survival time of HCC patients with cirrhosis and it is a confounding factor when cortactin was applied to predict survival time of patients.

Assessment of prognosis is a crucial step in management of patients with HCC. The BCLC strategy has been validated externally in prospective studies [41] and has been endorsed by several scientific associations [12]. Here the authors also analyzed the group of data which stratified by BCLC stage system to understand the significance of cortactin expression to the clinical stage. We simplified the BCLC stage into two stages: 0-B and C-D. Survival analysis stratified by simplified BCLC stage showed that in the stratum with BCLC stage 0-B, the median survival time was 63 ± 5.93 months in patients whose cortactin IHC score was 0–3, which was much longer than those whose IHC score was 4–6 (37 ± 12.73 months). The difference between the two IHC score levels was significant (*P* = 0.015) (Figure 11C). In stratum with BCLC stage C-D, the significant difference was not found between cortactin IHC score level of 0–3 and 4–6 (*P* >0.05) (Figure 11D). These results suggest that, cortactin examination can get more accurate prediction in very early, early, and intermediate HCC. But it may do not apply to advanced and terminal HCC cases. The possible reason is that BCLC stage C includes the cases that present vascular invasion or extrahepatic spread. This criterion consequentially weakens the power of cortactin forecasting role. Very interestingly, this weakening also demonstrates on the other hand that the high level of cortactin expression in HCC identifies a high capability of invasion and metastasis.

All the findings in our experiments support that cortactin and CTTN expression levels are correlated with the invasiveness and migration of HCC and give the evidence that overexpression of cortactin and CTTN mRNA contribute to HCC invasive and metastasis. Cortactin examination has more predictive power in patients with Child-Pugh grade A and BCLC stage 0-B. In addition to the satisfactorily predictive power to HCC prognosis, we also develop a valuable method that can precisely detect CTTN mRNA level with the advantage of sensitivity, specificity, and repeatability. This method can be used for other quantitative studies about CTTN expression. In conclusion, overexpression of cortactin is closely associated with poor prognosis in human HCC. Cortactin and CTTN should be used for differentiating varieties of survival for patients with HCC.

## Conclusion

Overexpression of cortactin is associated with high invasiveness of hepatoma carcinoma cells, which closely involved poor human HCCs prognosis that caused by cancer embolus and metastasis. Cortactin examination has more predictive power in patients with Child-Pugh grade A and BCLC stage 0-B. As a sensitive biomarker, cortactin should be used for differentiating varieties of survival for patients after HCC resection.

## Abbreviations

AFP: α-fetoprotein; Ct: Cycle values; DMEM: Dulbecco minimum essential medium; ECM: Extracellular matrix; FBS: Fetal bovine serum; GAPDH: Glyceraldehyde phosphate dehydrogenase; HCC: Hepatocellular carcinoma; IHC: Immunohistochemistry; N-WASP: Neural Wiskott-Aldrich syndrome protein; RMLN: Natural logarithm of relative magnitude; RT-PCR: Reverse transcription-polymerase chain reaction.

## Competing interests

The authors declare that they have no competing interests.

## Authors’ contributions

ZG was the main initiator of this work, plus wrote and edited the English; HZM carried out the cell biological experiments; KYL prepared the liver tissue samples; ZG and WDQ performed the molecular biological experiments and analysis. LY and RL completed the pathological diagnosis and classification; ZG and HZM performed clinical and empirical data recoding and analysis; ZHY took all responsibility and was the corresponding author. The authors take full responsibility for the scope, direction, and content of the manuscript and have approved the submitted manuscript.
